# Supplementary material for: Phage peptides mediate precision base editing with focused targeting window
Source: Nat Commun. 2022 Mar 29;13:1662. doi: 10.1038/s41467-022-29365-7 (PMC8964698; doi:10.1038/s41467-022-29365-7)
Supplement: Supplementary file 1 — Supplementary Information [file 41467_2022_29365_MOESM1_ESM.pdf]

Supplementary Information

Phage Peptides Mediate Precision Base Editing with Focused Targeting Window

Kun Jia[1,2,3,#], Yan-ru Cui[1,4,#], Shisheng Huang[1,4], Peihong Yu[1,4], Zhengxing Lian[5], Peixiang Ma[1], Jia Liu[1,2,3,6,7,*]

[1]Shanghai Institute for Advanced Immunochemical Studies and School of Life Science and Technology, ShanghaiTech University, Shanghai, 201210, China

[2]The State Kay Laboratory of Respiratory Disease, First Affiliated Hospital of Guangzhou Medical University, Guangzhou, 510120, China.

[3]Shanghai Clinical Research and Trial Center, Shanghai, 201210, China

[4]University of Chinese Academy of Sciences, Beijing, 100049, China

[5]Beijing Key Laboratory of Animal Genetic Improvement, China Agricultural University, 2 Yuanmingyuan West Rd., Haidian District, Beijing 100094, China

[6]Gene Editing Center, School of Life Science and Technology, ShanghaiTech University, 201210, China

[7]Guangzhou Laboratory, No. 9 XingDaoHuanBei Road, Guangzhou International Bio Island, Guangzhou 510005, Guangdong Province, China

[#]These authors contributed equally to this work..

*Correspondence should be addressed to J.L. (liujia@shanghaitech.edu.cn)

Table of Content

1. **Supplementary Experimental Procedure**

2. **Supplementary Figures**

3. **Supplementary Tables**

Supplementary Figures

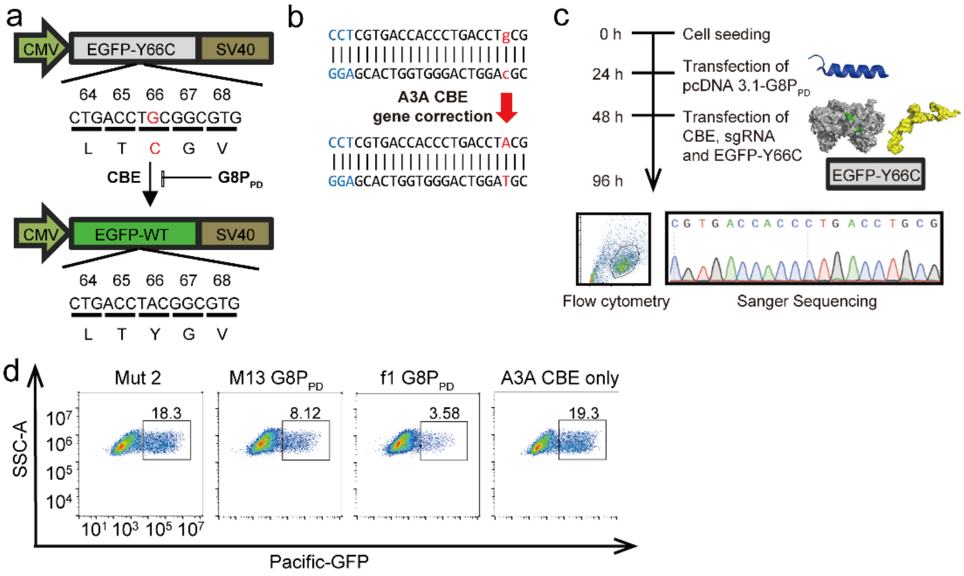

Supplementary Fig. 1 (related to Fig. 1): Evaluation of the inhibitory effects of G8Ps on CBE using transiently transfected EGFP reporter. a Schematic presentation of the EGFP reporter assay. Nucleotide and amino acid mutations are highlighted in red. **b** Design of sgRNA. PAM sequences and CBE-targeted nucleotides are highlighted in blue and red respectively. **c** Flow chart showing experimental procedures. The cartoon is blue for G8P$_{PD}$ peptide, grey for CBE and yellow for sgRNA, respectively. **d** Representative flow cytometry images for the EGFP reporter assay. The percentage of EGFP positive cells is indicated.

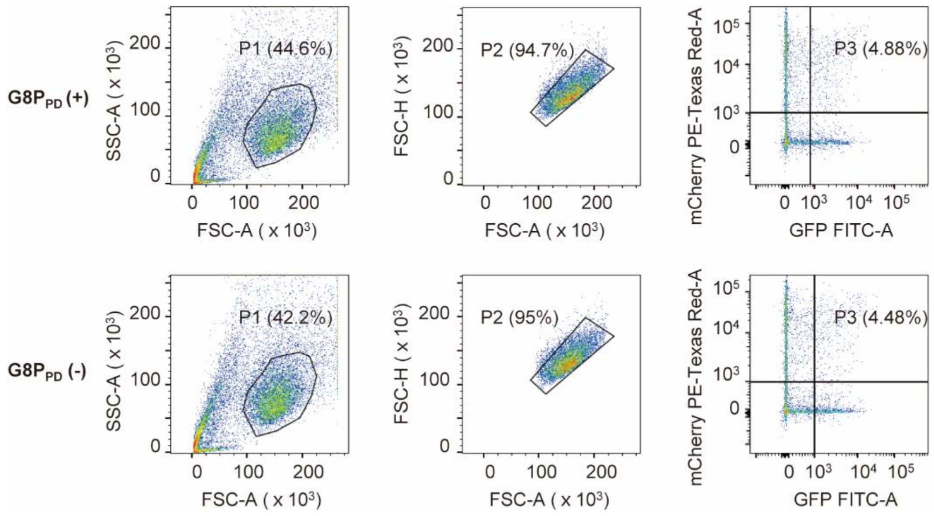

Supplementary Fig. 2: Sorting of GFP and mCherry dual positive cells by flow cytometry. The G8P$_{PD}$-free group is treated with an empty plasmid carrying mCherry gene to control for cell stress induced by flow cytometry and serial transfection.

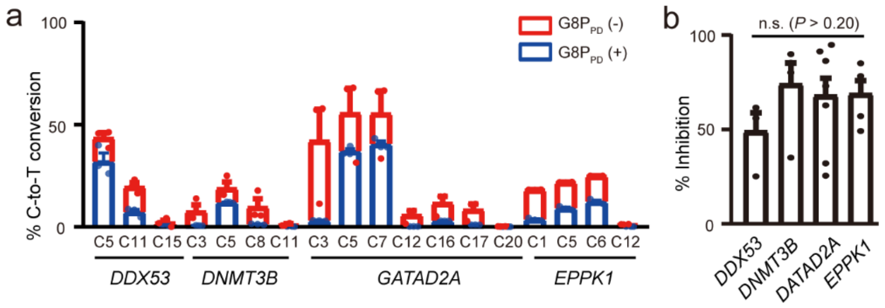

Supplementary Fig. 3 (related to Fig. 2): The inhibitory activities of f1 G8P$_{PD}$ to A3A CBE-induced C-to-T conversion in HEK293T cells. a NGS analyses of A3A CBE-induced C-to-T conversion in the absence and presence of f1 G8P$_{PD}$. **b** Comparison of position-averaged inhibition rates of G8P$_{PD}$ between different target sites. The difference between every two groups is not significant, as determined by two-tailed Student's *t* test. For **a-b**, the data are shown as mean ± standard error of mean (SEM) (*n* = 3 biologically independent replicates).

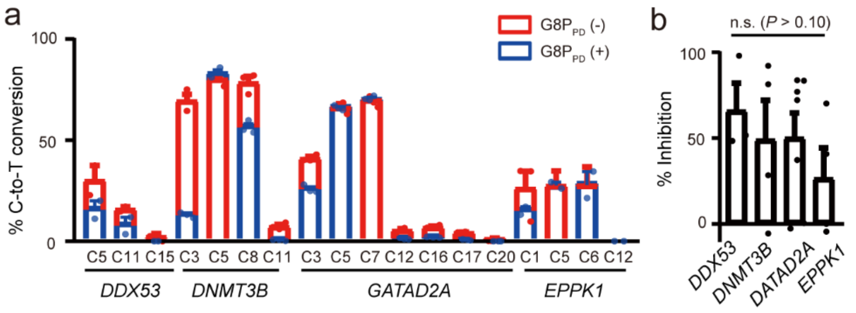

Supplementary Fig. 4 (related to Fig. 2): The inhibitory activities of f1 G8P$_{PD}$ to A3A CBE-induced C-to-T conversion in U-2 OS cells. a NGS analyses of A3A CBE-induced C-to-T conversion in the absence and presence of f1 G8P$_{PD}$. **b** Comparison of position-averaged inhibition rates of G8P$_{PD}$ between different target sites. The difference between every two groups is not significant, as determined by two-tailed Student's *t* test. For **a-b**, the data are shown as mean ± SEM. *DDX53* and *EPPK1* groups contain 2 biologically independent replicates and *DNMT3B* and *GATAD2A* groups contain 3 biologically replicates.

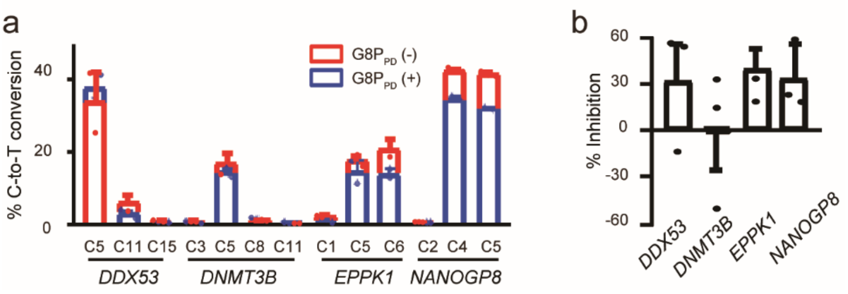

Supplementary Fig. 5 (related to Fig. 3): The inhibitory activities of f1 G8P$_{PD}$ to BE3 CBE-induced C-to-T conversion in HEK293T cells. a NGS analyses of BE3 CBE-induced C-to-T conversion in the absence and presence of f1 G8P$_{PD}$. **b** Comparison of position-averaged inhibition rates of G8P$_{PD}$ between different target sites. For **a-b**, the data in this figure are shown as mean ± SEM. *DDX53*, *DNMT3B* and *EPPK1* groups contain 2 biologically independent replicates and *NANOGP8* groups contain 3 biologically replicates.

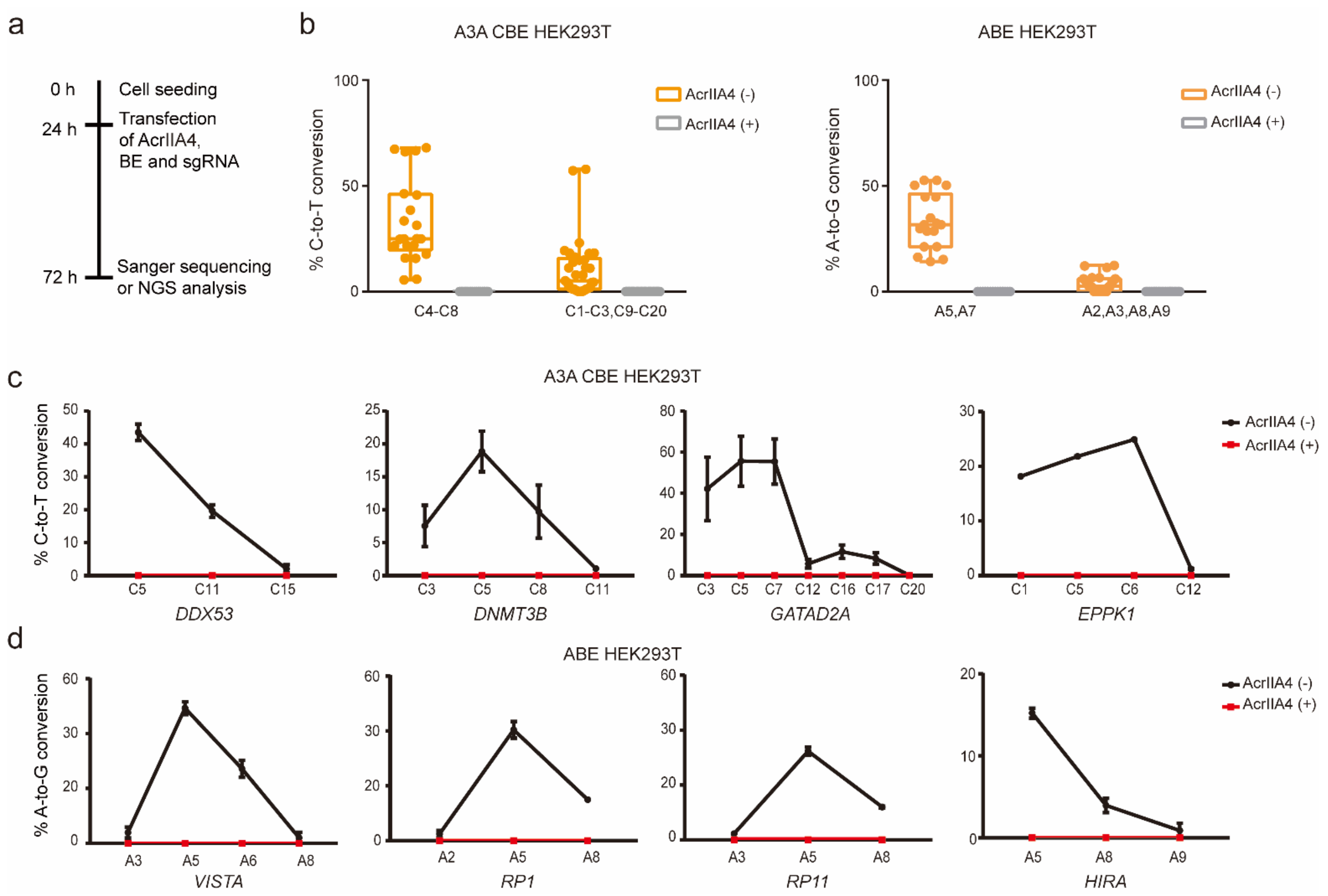

Supplementary Fig. 6: Analyses of the effects of co-transfected AcrIIA4 on the base-editing activity of CBE and ABE. a Flow chart showing experimental procedures. **b** The inhibitory activity of AcrIIA4 at on-target and out-of-window editing positions of A3A CBE and ABE in HEK293T cells. The center line in each box indicates the median. The lower and upper bounds of each box represent the first quartile (25%) and the third quartile (75%), respectively. The bottom and top of whiskers denote the minimum and maximum, respectively. **c** AcrIIA4 abolishes the activity of A3A CBE at designated genomic loci, as determined by NGS analyses. **d** AcrIIA4 abolishes the activity of ABE at designated genomic loci, as determined by NGS analyses. For **c-d**, the data are shown as mean ± SEM. For **b-d**, 3 biologically independent replicates are performed.

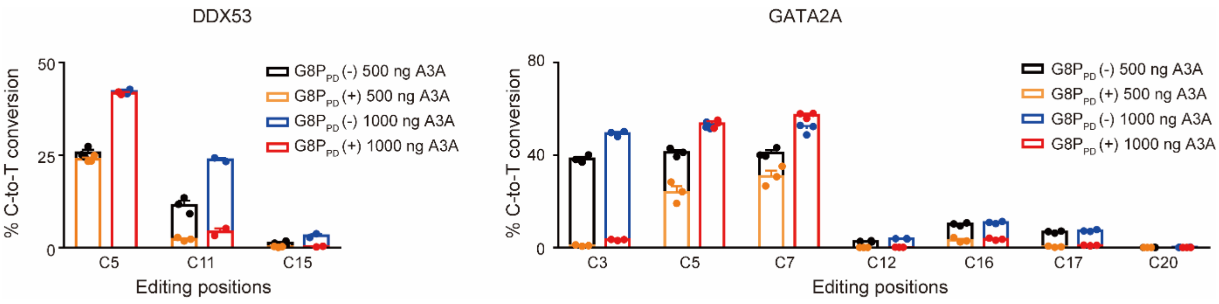

Supplementary Fig. 7: The effects of plasmid dosage and G8P$_{PD}$ on the editing activity of A3A BE3. sgRNA plasmids were fixed to 250 ng. The data are shown as mean ± SEM (*n* = 3 biologically independent replicates).

A3A CBE -G8P$_{PD}$ Rep #1	Leu			Arg			Val			Asn			Thr			Ile			Gly			% Reads
	C1	T	A	C4	G	T	G	T	T	A	A	C12	A	C14	C15	A	T	T	G	G	*FBN1*^{T7498C}	18.4
	C1	T	A	T4	G	T	G	T	T	A	A	C12	A	C14	C15	A	T	T	G	G	WT	3.8
	T1	T	A	T4	G	T	G	T	T	A	A	C12	A	C14	C15	A	T	T	G	G	Type 1	33.0
	T1	T	A	T4	G	T	G	T	T	A	A	T12	A	C14	C15	A	T	T	G	G	Type 2	3.2
	T1	T	A	T4	G	T	G	T	T	A	A	T12	A	T14	T15	A	T	T	G	G	Type 3	1.7
	T1	T	A	T4	G	T	G	T	T	A	A	C12	A	C14	T15	A	T	T	G	G	Type 4	1.7

A3A CBE -G8P$_{PD}$ Rep #2	Leu			Arg			Val			Asn			Thr			Ile			Gly			% Reads
	C1	T	A	C4	G	T	G	T	T	A	A	C12	A	C14	C15	A	T	T	G	G	*FBN1*^{T7498C}	24.5
	C1	T	A	T4	G	T	G	T	T	A	A	C12	A	C14	C15	A	T	T	G	G	WT	4.8
	T1	T	A	T4	G	T	G	T	T	A	A	C12	A	C14	C15	A	T	T	G	G	Type 1	27.0
	T1	T	A	T4	G	T	G	T	T	A	A	T12	A	C14	C15	A	T	T	G	G	Type 2	5.8
	C1	T	A	T4	G	T	G	T	T	A	A	T12	A	C14	T15	A	T	T	G	G	Type 3	1.0

A3A CBE -G8P$_{PD}$ Rep #3	Leu			Arg			Val			Asn			Thr			Ile			Gly			% Reads
	C1	T	A	C4	G	T	G	T	T	A	A	C12	A	C14	C15	A	T	T	G	G	*FBN1*^{T7498C}	18.6
	C1	T	A	T4	G	T	G	T	T	A	A	C12	A	C14	C15	A	T	T	G	G	WT	2.3
	T1	T	A	T4	G	T	G	T	T	A	A	C12	A	C14	C15	A	T	T	G	G	Type 1	30.4
	T1	T	A	T4	G	T	G	T	T	A	A	T12	A	C14	C15	A	T	T	G	G	Type 2	4.8
	C1	T	A	T4	G	T	G	T	T	A	A	T12	A	C14	T15	A	T	T	G	G	Type 3	1.0
	T1	T	A	T4	G	T	G	T	T	A	A	T12	A	C14	T15	A	T	T	G	G	Type 4	2.2

A3A CBE +G8P$_{PD}$ Rep #1	Leu			Arg			Val			Asn			Thr			Ile			Gly			% Reads
	C1	T	A	C4	G	T	G	T	T	A	A	C12	A	C14	C15	A	T	T	G	G	*FBN1*^{T7498C}	21.9
	C1	T	A	T4	G	T	G	T	T	A	A	C12	A	C14	C15	A	T	T	G	G	WT	38.3
	T1	T	A	T4	G	T	G	T	T	A	A	C12	A	C14	C15	A	T	T	G	G	Type 1	16.5
	T1	T	A	C4	G	T	G	T	T	A	A	C12	A	C14	C15	A	T	T	G	G	Type 2	1.1

A3A CBE +G8P$_{PD}$ Rep #2	Leu			Arg			Val			Asn			Thr			Ile			Gly			% Reads
	C1	T	A	C4	G	T	G	T	T	A	A	C12	A	C14	C15	A	T	T	G	G	*FBN1*^{T7498C}	28
	C1	T	A	T4	G	T	G	T	T	A	A	C12	A	C14	C15	A	T	T	G	G	WT	36.0
	T1	T	A	T4	G	T	G	T	T	A	A	C12	A	C14	C15	A	T	T	G	G	Type 1	12.5
	C1	T	A	T4	G	T	G	T	T	A	A	C12	A	T14	C15	A	T	T	G	G	Type 2	1.1

A3A CBE +G8P$_{PD}$ Rep #3	Leu			Arg			Val			Asn			Thr			Ile			Gly			% Reads
	C1	T	A	C4	G	T	G	T	T	A	A	C12	A	C14	C15	A	T	T	G	G	*FBN1*^{T7498C}	26.1
	C1	T	A	T4	G	T	G	T	T	A	A	C12	A	C14	C15	A	T	T	G	G	WT	41.9
	T1	T	A	T4	G	T	G	T	T	A	A	C12	A	C14	C15	A	T	T	G	G	Type 1	10.9
	T1	T	A	C4	G	T	G	T	T	A	A	C12	A	C14	C15	A	T	T	G	G	Type 2	1.2

Supplementary Fig. 8 (related to Fig. 4): The effects of G8P$_{PD}$ on A3A CBE-mediated gene correction of *FBN1*^{T7498C} mutation. Perfectly and imperfectly edited alleles are shown as percentage of total reads.

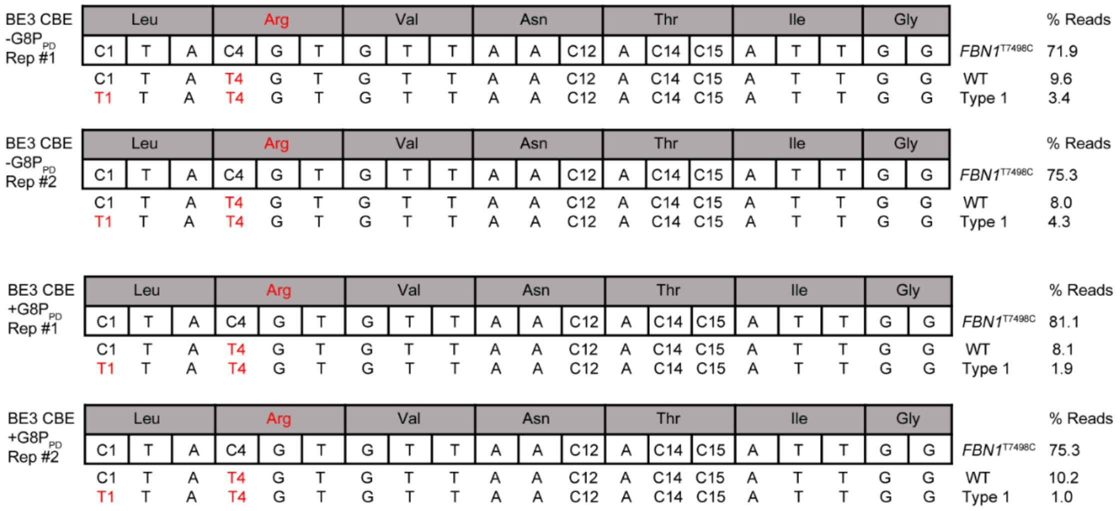

Supplementary Fig. 9 (related to Fig. 4): The effects of G8P$_{PD}$ on BE3 CBE-mediated gene correction of *FBN1*^{T7498C} mutation. Perfectly and imperfectly edited alleles are shown as percentage of total reads.

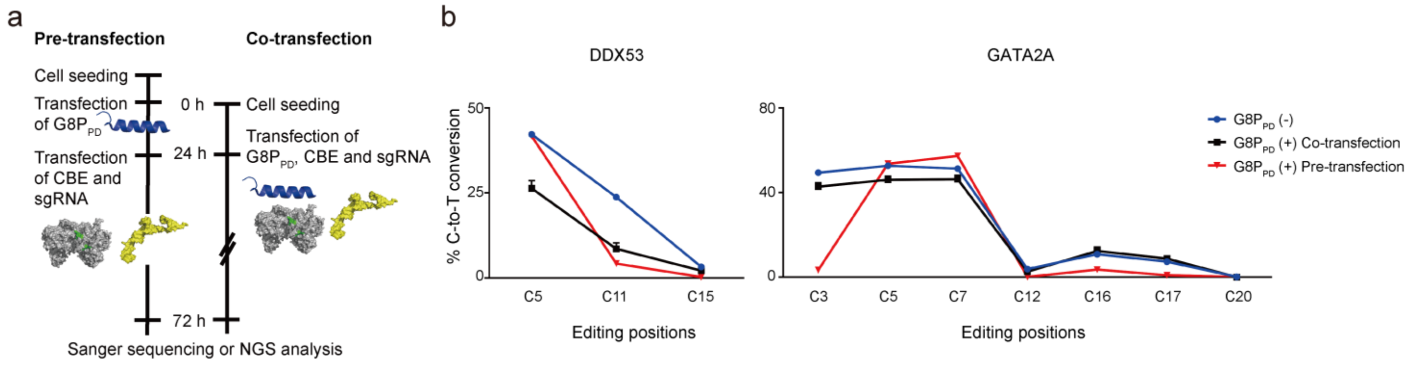

Supplementary Fig. 10: G8P$_{PD}$ pre-transfection, but not co-transfection, improves the targeting specificity of A3A CBE. a Flow chart illustrating experimental procedures. **b** Analysis of the effects of pre-transfected and co-transfected G8P$_{PD}$ on the editing activity of A3A CBE at *DDX53* and *GATA2A* sites. The data are shown as mean ± SEM (*n* = 2 biologically independent replicates).

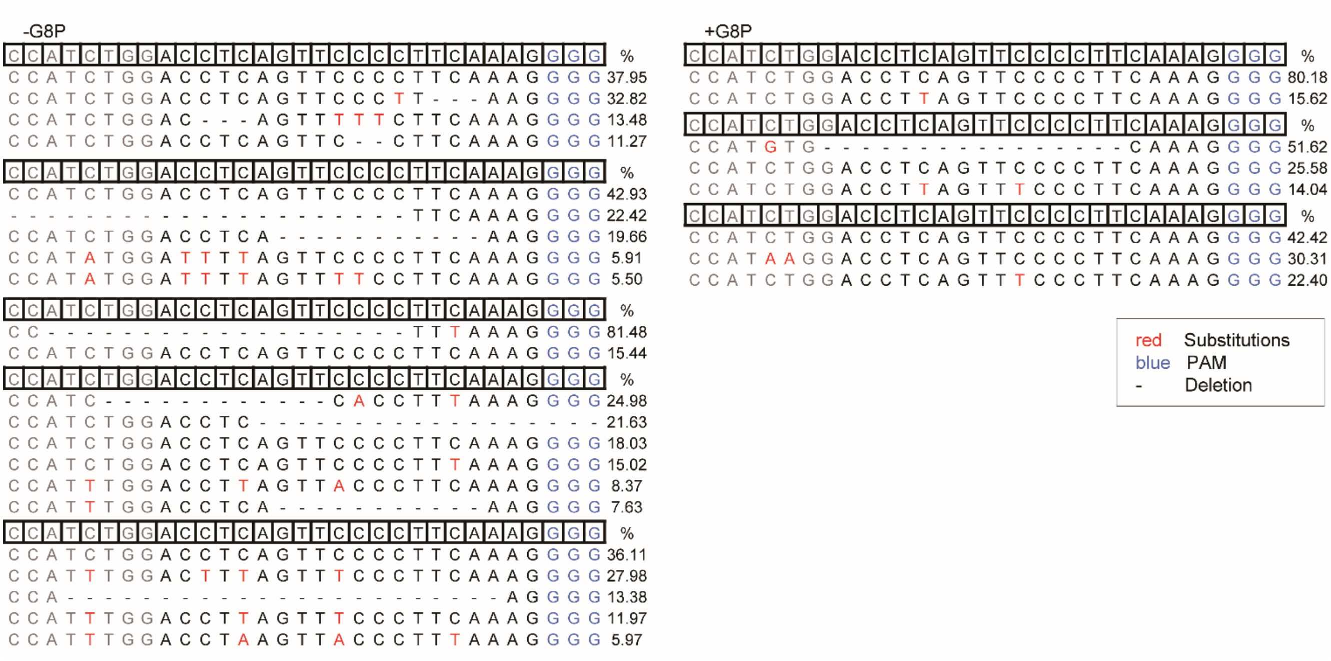

Supplementary Fig. 11 (related to Fig. 5): The effects of G8P$_{PD}$ mRNA on A3A CBE plasmid-mediated base editing (microinjection DNA group) in mouse embryos. Edited alleles are shown as percentage of total reads.

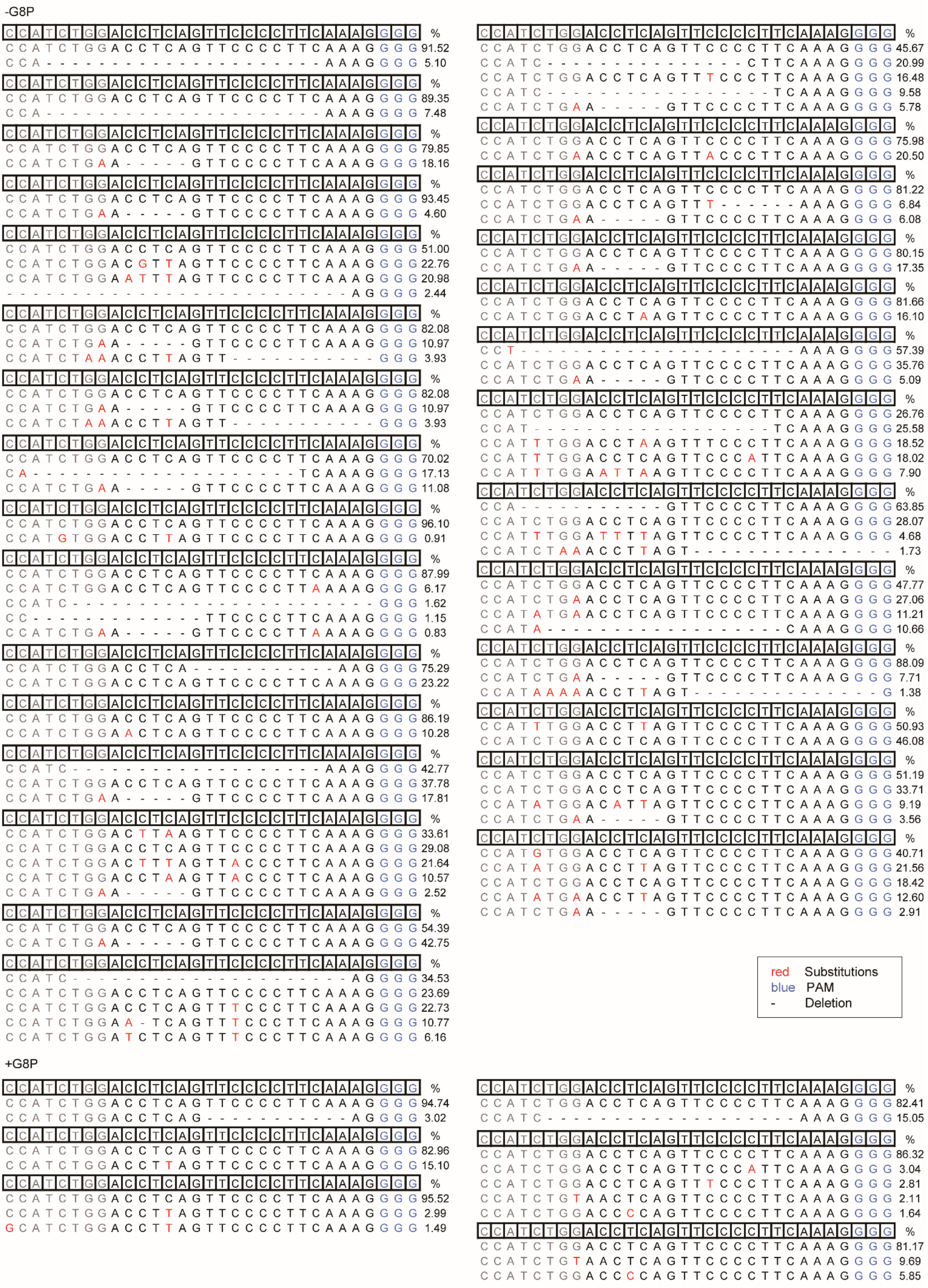
-G8P
+G8P
red Substitutions
blue PAM
- Deletion

Supplementary Fig. 12 (related to Fig. 5): The effects of G8P peptide on A3A and sgRNA RNA-mediated base editing (microinjection RNA group) in mouse embryos. Edited alleles are shown as percentage of total reads.

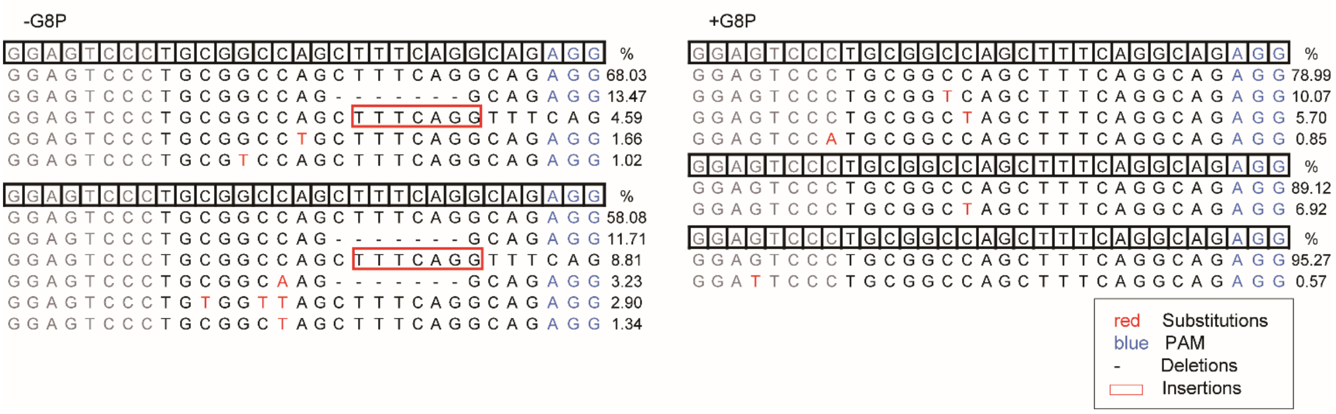

Supplementary Fig. 13 (related to Fig. 5): The effects of G8P peptide on BE3 and sgRNA RNA-mediated base editing (microinjection RNA group) in mouse embryos. Edited alleles are shown as percentage of total reads.

Supplementary Tables

Supplementary Table 1. BE-targeted genomic sites (PAM sequences uppercased)

**Genes**	**Sequence (5' to 3')**
DNMT3B	ggcactgcggctggaggtggGGG
GATAD2A	ggcactcagaacattcctgcTGG
EPPK1	cgtaccgagagctgcaggagAGG
DDX53	tgatcaagagcgagcagtagAGG
VISTA	gaacacaaagcatagactgcGGG
RP1	gagtatgaggcatagactgcAGG
RP11	gaatactaagcatagactccAGG
HIRA	gaagaccaaggatagactgcTGG
NANOGP8	gctccaggactggatgttctGGG
FBN1^{T7498C}	ctacgtgttaacaccattggCGG
Tyr-1	acctcagttccccttcaaagGGG
Tyr-2	tgcggccagctttcaggcagAGG

Supplementary Table 2. Sequences of oligonucleotides for sgRNA synthesis

sgRNA	Upstream (5' to 3')	Downstream (5' to 3')
sgDNMT3B	accgggcactgcggctggaggtgg	aaacccacctccagccgcagtgcc
sgGATAD2A	accgggcactcagaacattcctgc	aaacgcaggaatgttctgagtgcc
sgEPPK1	accgcgtaccgagagctgcaggag	aaacctcctgcagctctcggtacg
sgDDX53	accgtgatcaagagcgagcagtag	aaacctactgctcgctcttgatca
sgVISTA	accgaacacaaagcatagactgc	aaaccagtctatgctttgtgttc
sgRP1	accgagtatgaggcatagactgc	aaactcgcagtctatgcctcatactc
sgRP11	accgaatactaagcatagactcc	aaacctggagtctatgcttagtattc
sgHIRA	accgcagtctatccttggtcttc	aaacaagaccaaggatagactgc
sgFBN1	accgctacgtgttaacaccattgg	aaacccaatggtgttaacacgtga
sgNANOGP8	accggctccaggactggatgttct	aaacgaacatccagtcctggagc
Tyr-sg1	accgacctcagttccccttcaaag	aaacctttgaaggggaactgaggt
Tyr-sg2	accgtgcggccagctttcaggcag	aaacctgcctgaaagctggccgca

Supplementary Table 3. Sanger sequencing primers

Genes	Forward (5' to 3')	Reverse (5' to 3')
DNMT3B	agcaaatatgtaagtcccaggtca	tgggggatcagaagccctaa
GATAD2A	atgccgtagaggaggaagac	tgagtgagtcccaccaacg
EPPK1	cacaggagacacgatcccg	cccgtctggtgtgtgttcta
DDX53	gctgatgacttgtcaagcgac	cagtctttcctgtccgtcca
VISTA	gtggtaattttccagcccgc	gtccagccccatctgtcaaa
RP1	gcttttcagctagagggaacct	tgaaatgctgtgcgtgtct
RP11	gatgccctccatcttctccg	taggtttgcatagacctgccc
HIPA	cctccatcttcctacacgcc	gaacaggcagcgtattgctt
FBN1^{T7498C}	caggacgtatggtgttgggt	ccctatgaagcagctccacg
NANOGP8	acagacagttctggttgtcct	ggactgttccaggcctgatt
Tyr	atggatgggtgatgggagtc	catgaagttgcctgagcactg

Supplementary Table 4. Deep sequencing primers

Gene	Forward (5' to 3')	Reverse (5' to 3')
DNMT3B	gtccaaagcaggatgacagg	agacgtccaaaaccagactcc
GATAD2A	acaggttctgttgggagcac	ggaaagcgcaacctcttgcag
EPPK1	tggaaacagcctacagacgg	tctcgtcatcgatgtgctcg
DDX53	gctgatgacttgtcaagcgac	cagtctttcctgtccgtcca
VISTA	gtggtaattttccagcccgc	gtccagccccatctgtcaaa
RP1	gcttttcagctagagggaacct	tgaaatgctgtgcgtgtct
RP11	gatgccctccatcttctccg	taggtttgcatagacctgccc
HIPA	cctccatcttcctacacgcc	gaacaggcagcgtattgctt
FBN1^{T7498C}	caggacgtatggtgttgggt	ccctatgaagcagctccacg
NANOGP8	acagacagttctggttgtcct	ggactgttccaggcctgatt
Tyr	atggatgggtgatgggagtc	catgaagttgcctgagcactg
P5 primer	aatgatacggcgaccaccgagatctacac	
P7 primer	caagcagaagacggcatacgagat	
