# Supplementary material for: Phage peptides mediate precision base editing with focused targeting window
Source: Nat Commun. 2022 Mar 29;13:1662. doi: 10.1038/s41467-022-29365-7 (PMC8964698; doi:10.1038/s41467-022-29365-7)
Supplement: Supplementary file 2 — Reporting Summary [file 41467_2022_29365_MOESM2_ESM.pdf]

Corresponding author(s): Jia Liu

Last updated by author(s): Mar 10, 2022

Reporting Summary

Nature Research wishes to improve the reproducibility of the work that we publish. This form provides structure for consistency and transparency in reporting. For further information on Nature Research policies, see our Editorial Policies and the Editorial Policy Checklist.

Statistics

For all statistical analyses, confirm that the following items are present in the figure legend, table legend, main text, or Methods section.

n/a	Confirmed	
☐	☒	The exact sample size (n) for each experimental group/condition, given as a discrete number and unit of measurement
☐	☒	A statement on whether measurements were taken from distinct samples or whether the same sample was measured repeatedly
☐	☒	The statistical test(s) used AND whether they are one- or two-sided<br>*Only common tests should be described solely by name; describe more complex techniques in the Methods section.*
☒	☐	A description of all covariates tested
☒	☐	A description of any assumptions or corrections, such as tests of normality and adjustment for multiple comparisons
☐	☒	A full description of the statistical parameters including central tendency (e.g. means) or other basic estimates (e.g. regression coefficient) AND variation (e.g. standard deviation) or associated estimates of uncertainty (e.g. confidence intervals)
☐	☒	For null hypothesis testing, the test statistic (e.g. F, t, r) with confidence intervals, effect sizes, degrees of freedom and P value noted<br>*Give P values as exact values whenever suitable.*
☒	☐	For Bayesian analysis, information on the choice of priors and Markov chain Monte Carlo settings
☒	☐	For hierarchical and complex designs, identification of the appropriate level for tests and full reporting of outcomes
☒	☐	Estimates of effect sizes (e.g. Cohen's d, Pearson's r), indicating how they were calculated

Our web collection on statistics for biologists contains articles on many of the points above.

Software and code

Policy information about availability of computer code

Data collection	Illumina HiSeq Control Software (HCS), OLB and GAPipeline-1.6 were used on the Illumina Hiseq sequencers to collect the next-generation sequencing (NGS) data. Illumina Miseq control software (3.1) was used on the Illumina Miseq sequencers to collect the high-throughput sequencing data.
Data analysis	Flow cytometry analysis was performed by Beckman Coulter CytFLEX (Beckman Coulter, Brea, USA) and cell sorting was performed using BD FACSAria III flow cytometry (BD Biosciences, New York, USA). FlowJo VX or FlowJo 10 was used to analyze flow cytometry data. Analyses of the Sanger sequencing results of the PCR product of edited EGFP reporter were performed using EditR 1.0.10. When NGS data were analyzed, amplicons with less than 6 M read counts were excluded and the adapter sequence of the paired-end reads were removed using AdapterRemoval 2.2.2. All processed reads were then mapped to the target sequences using the BWA-MEM algorithm (BWA v0.7.16). The mutation rate was calculated using bam-readcount 0.8.0 with parameters -q 20 -b 30. Three biological replicates were processed by using Illumina HiSeq 3000. Frequency, mean, and standard error of mean (SEM) were calculated using GraphPad Prism 8. Statistical analyses were performed using two-tailed Student’s t test unless otherwise noted.

For manuscripts utilizing custom algorithms or software that are central to the research but not yet described in published literature, software must be made available to editors and reviewers. We strongly encourage code deposition in a community repository (e.g. GitHub). See the Nature Research guidelines for submitting code & software for further information.

Data

Policy information about availability of data

All manuscripts must include a data availability statement. This statement should provide the following information, where applicable:

- Accession codes, unique identifiers, or web links for publicly available datasets
- A list of figures that have associated raw data
- A description of any restrictions on data availability

NGS data have been deposited in the NCBI Sequence Read Archive database under the accession code SRP312256 (cell lines; https://www.ncbi.nlm.nih.gov/sra/?term=SRP312256) and PRJNA798574 (embryos; https://www.ncbi.nlm.nih.gov/sra/?term=PRJNA798574). Source data are provided with this paper.

Field-specific reporting

Please select the one below that is the best fit for your research. If you are not sure, read the appropriate sections before making your selection.

[x] Life sciences [ ] Behavioural & social sciences [ ] Ecological, evolutionary & environmental sciences

For a reference copy of the document with all sections, see nature.com/documents/nr-reporting-summary-flat.pdf

Life sciences study design

All studies must disclose on these points even when the disclosure is negative.

Sample size	Two or three biological replicates were included for each experimental condition. In practice, three biological replicates were performed and samples sent for NGS analysis. Accidentally, problems may occur leading to loss of samples during sample preparation or sequencing. In these cases, if two replicates could be obtained then the data will be collected. Otherwise, the whole experiments will be repeated to keep consistency between parallel groups. The exact number of samples are reflected in each figure by the number of data points and in the legends. We have revised the Methods section to clearly indicate this.
Data exclusions	No data were excluded.
Replication	Two or three biological replicates were performed. For some replicate attempts, technical problems may occur, but none of the obtained data were excluded. Significant difference was determined as described in the text.
Randomization	All individual mice or samples were randomly allocated to the experimental groups.
Blinding	The study was essentially not blinded because it was necessary for investigators to be aware of the information of mouse genotypes and cell types to be compared.

Reporting for specific materials, systems and methods

We require information from authors about some types of materials, experimental systems and methods used in many studies. Here, indicate whether each material, system or method listed is relevant to your study. If you are not sure if a list item applies to your research, read the appropriate section before selecting a response.

Materials & experimental systems

n/a	Involved in the study	
[x]	[ ]	Antibodies
[ ]	[x]	Eukaryotic cell lines
[x]	[ ]	Palaeontology and archaeology
[ ]	[x]	Animals and other organisms
[x]	[ ]	Human research participants
[x]	[ ]	Clinical data
[x]	[ ]	Dual use research of concern

Methods

n/a	Involved in the study	
[x]	[ ]	ChIP-seq
[ ]	[x]	Flow cytometry
[x]	[ ]	MRI-based neuroimaging

Eukaryotic cell lines

Policy information about cell lines

Cell line source(s)	ATCC: U2-OS, HEK293T
Authentication	Cell ID was determined by VivaCell Biosciences using STR profiling.
Mycoplasma contamination	No contamination was found
Commonly misidentified lines (See ICLAC register)	None used

Animals and other organisms

Policy information about studies involving animals; ARRIVE guidelines recommended for reporting animal research

Laboratory animals	Female C57BL/6 mice of 3.5 to 4 weeks old and male C57BL/6 mice of 3 to 6 months old were used in our experiments.
Wild animals	No wild animals were used in the study.
Field-collected samples	No field-collected samples were used in the study.
Ethics oversight	The use and care of animals were complied with the guidelines of the Institutional Animal Care and Use Committee (IACUC) of GemPharmatech Co., Ltd, Nanjing, Jiangsu, China. Mice were maintained in a specific pathogen-free (SPF) facility under a 12 h dark-light cycle.

Note that full information on the approval of the study protocol must also be provided in the manuscript.

Flow Cytometry

Plots

Confirm that:

- [x] The axis labels state the marker and fluorochrome used (e.g. CD4-FITC).
- [x] The axis scales are clearly visible. Include numbers along axes only for bottom left plot of group (a 'group' is an analysis of identical markers).
- [x] All plots are contour plots with outliers or pseudocolor plots.
- [x] A numerical value for number of cells or percentage (with statistics) is provided.

Methodology

Sample preparation	Cells were resuspended at 2*10^5cells/200μL in PBS supplemented with 2% fetal calf serum (FCS buffer) and filtered through a 70-μm strainer, Data for at least 2000 cells were collected.
Instrument	Beckman Coulter CytoFLEX; BD FACSAria III
Software	Beckman Coulter CytExpert2.0.0.153; BD FACSDiva 8.0.1
Cell population abundance	For flow cytometry analysis,Of the surviving HEK293T cells analyze FITC fluorescence positive ; For cell sorting ,Of the surviving single sorted HEK 293T cells FITC and PE-Texas Red fluorescence double positive cells were collected ,cells were genotyped using next-generation sequencing(illumina)
Gating strategy	For flow cytometry analysis , HEK 293T cells were initially gated on population using SSC-A FSC-A (Gate P1) and then analyzed for singlets using SSC-A/SSC-H (Gate P2).Live cells were analyzed % parent for by gating for FITC-positive cells(P3); For cell sorting, HEK293T and U2OS cells were initially gated on population using FSC-H/FSC-A (Gate P1) and then sorted for singlets using SSC-A/SSC-H (Gate P2).Live cells were sorted for by gating for FITC and PE-Texas Red fluorescence-dual positive cells (Q2),P1 represented starting cell population, P3 represented aimed cell population, FSC gating for FITC(Q3) , and SSC gating for PE-Texas Red fluorescence(Q4) , Q2 of FITC and PE-Texas Red fluorescence represented collected cells.

- [x] Tick this box to confirm that a figure exemplifying the gating strategy is provided in the Supplementary Information.
